# Post-traumatic stress disorder prevalence and subtypes among survivors of critical illness

**DOI:** 10.1186/cc14635

**Published:** 2015-03-16

**Authors:** M Patel, J Jackson, A Morandi, T Girard, C Hughes, A Kiehl, J Thompson, R Chandrasekhar, E Ely, P Pandharipande

**Affiliations:** 1 Vanderbilt University, Nashville, TN, USA; 2 Ancelle Hospital, Cremona, Italy

## Introduction

Among North American survivors of critical illness, we aim to describe the prevalence of post-traumatic stress disorder (PTSD), and its subtypes of intrusion, avoidance, and hyperarousal.

## Methods

In this prospective, observational, multicenter cohort study from 2009 to 2010, we screened adults (age ≥18 years) with new-onset respiratory failure, cardiogenic shock, or septic shock, who were admitted to medical and surgical ICUs in four facilities. At 3-month and 12-month follow-ups, high probability of PTSD was defined by 17-symptom PTSD Checklist - Event Specific Version (PCL-S) score ≥50. Also, PCL-S responses were mapped onto DSM-IV criteria for PTSD. To augment PTSD identification, those with a moderate probability of post-ICU PTSD (PCL-S score ≥35) were further confirmed with the Clinician Administered PTSD Scale (CAPS) structured interview. Moderate or greater symptoms for each PTSD subtype of intrusion, avoidance, and hyperarousal were categorized.

## Results

Of the 180 eligible participants at 3 months, PTSD was identified in 10 (6%) using PCL-S scores and 15 (8%) using DSM-IV mapping of the PCL-S. Of the 160 eligible participants at 12 months, PTSD was identified in two (1%) using PCL-S scores and 10 (6%) using DSM-IV mapping of the PCL-S. Of those eligible for CAPS assessments, at 3 months only 13 of 24 (54%) interviews were completed resulting in three extra PTSD diagnoses, and at 12 months only six of 22 (27%) interviews were completed resulting in two extra PTSD diagnoses. At 3 and 12 months, the intrusion subtype was present in 25 (14%) and in 25 (16%), the avoidance subtype was present in 78 (43%) and 60 (38%), and the hyperarousal subtype was present in 82 (46%) and 71 (44%).

## Conclusion

Irrespective of definition using PCL-S or DSM-IV mapping, PTSD was identified in no more than one in 10 survivors of critical illness at either 3 or 12 months post ICU, which is still nearly double the US population past-year PTSD prevalence. In ICU survivors with moderate probability PTSD by PCL-S, the CAPS gold-standard interview is challenging to complete and adds only a small number of diagnoses. However, two in five ICU survivors will develop PTSD subtypes of avoidance or hyperarousal, which both occur twice as frequently as the intrusion subtype. Targeting predominant PTSD subtypes may help optimize treatment strategies for the ICU survivor, such as prolonged exposure and eye movement desensitization and reprocessing for those with the avoidance subtype, and pharmacologic antidepressants targeting the sympathetic nervous system to produce anxiolysis for those with the hyperarousal subtype.

